# A Randomized Controlled Trial of a Research-Tested Mobile Produce Market Model Designed to Improve Diet in Under-Resourced Communities: Rationale and Design for the Veggie Van Study

**DOI:** 10.3390/ijerph19169832

**Published:** 2022-08-10

**Authors:** Leah N. Vermont, Christina Kasprzak, Anne Lally, Alicia Claudio, Laurene Tumiel-Berhalter, Lindsey Haynes-Maslow, Alice Ammerman, Samina Raja, Lucia A. Leone

**Affiliations:** 1 Department of Community Health and Health Behavior, School of Public Health and Health Professions, University at Buffalo, Buffalo, NY 14214, USA; 2 Department of Family Medicine, Jacobs School of Medicine and Biomedical Sciences, University at Buffalo, Buffalo, NY 14203, USA; 3 Department of Agricultural and Human Sciences, North Carolina State University, Raleigh, NC 27695, USA; 4 Department of Nutrition, Gillings School of Global Public Health, University of North Carolina at Chapel Hill, Chapel Hill, NC 27599, USA; 5 Department of Urban and Regional Planning, School of Architecture and Planning, University at Buffalo, Buffalo, NY 14214, USA

**Keywords:** mobile produce market, fruit and vegetable, food access, food environment, randomized control trial, dietary intake

## Abstract

Mobile produce markets are increasingly popular retail vendors used for providing access to fresh fruits and vegetables (F&V) in under-resourced communities; however, evaluation is limited due to design and implementation challenges. This protocol presents the original design of a randomized control trial aimed at assessing the effectiveness of the evidence-based Veggie Van (VV) mobile market model. Nine US community partner organizations were asked to partner with four community sites serving lower-income areas. Sites are randomized to either intervention or control. Intervention sites will host a mobile market for one year while the control sites will host planning events, with the goal to open a market afterward. Eligible participants are aged ≥ 18, the primary household shopper, live nearby/regularly frequent the site, and have expressed interest in learning about a mobile market. The primary outcome, F&V consumption, will be assessed via dietary recall at baseline and 12 months and compared between the intervention and control sites. This research advances work on the VV model and methods for mobile market evaluation with the addition of more robust measures and the study design. Determining the effectiveness of the VV model is imperative to justify taking it to scale to enhance the impact of mobile markets.

## 1. Introduction

The inability to access fresh fruits and vegetables (F&V) is a pervasive public health concern that is predominately affecting under-resourced communities; moreover, it is associated with numerous detrimental health and socioeconomic impacts. Individuals from racial/ethnic minority backgrounds and those who experience lower incomes are disproportionately impacted by poor nutrition and diet-related health outcomes, such as obesity, type 2 diabetes, hypertension, heart disease, stroke, and some cancers [1,2]. Lack of affordable, healthy food access and food insecurity are believed to be important contributing factors to these disparities. Mobile produce markets (mobile markets) are temporary food markets that regularly visit under-resourced communities and sell affordable, fresh, healthy food (e.g., F&V) [3]. Mobile markets are showing promise as effective food access interventions for mitigating these health disparities because they bring low-cost, healthy food directly to under-resourced populations [4,5,6,7].

A randomized control trial (RCT) demonstrated that the Veggie Van (VV) model, a food access model intended for mobile produce markets, is efficacious in increasing F&V consumption among lower-income customers [7]. The VV model is designed to address multiple dimensions of access to fresh produce by offering a variety of fresh, high-quality fruits and vegetables at a reduced cost. Produce is sold at convenient locations (i.e., places within a community) that are already serving the target population of families experiencing lower income through complementary services (i.e., health clinics, community centers, libraries etc.). A key component of the Veggie Van model is to encourage customers to purchase a bundle of produce (multiple items for a set price) rather than just one or two items separately. Cooking demonstrations, recipes, and nutritional education are also available at the market to help customers better use the produce in their bundles.

The current study aims to improve upon our prior work in multiple ways. First, we utilize more robust data collection methods of F&V consumption and purchasing, including a 24-h dietary recall, coupled with an objective indicator of F&V consumption (e.g., skin carotenoids measured via a finger scan), and the use of a standardized point of sale (POS) software to capture F&V purchasing. Second, the study seeks to increase the scale of the evaluation through implementation in multiple communities across the US. Lastly, this project was designed to allow mobile market partners to have greater involvement in the recruitment of community members into the RCT as well as the implementation of the VV model.

This paper will report on the methods deployed to reach the overall goal of this effectiveness–implementation study, which is to understand if mobile markets that implement the VV model can impact F&V intake and other related health outcomes in lower-income and under-resourced communities. The results of this study will be presented in a future publication.

## 2. Materials and Methods

### 2.1. Overview of Study Design

The goal of the VV study is to evaluate the effectiveness of mobile produce markets through a 12-month cluster-randomized controlled trial in 32 communities. The primary outcome measure is the F&V intake (cups/day) measured via a 24-h dietary recall. We utilized a request-for-partners (RFP) process to recruit organizations as study partners; the details of this process are reported elsewhere [8]. Briefly, this process involved broadly disseminating an RFP announcement eliciting interest among organizations that were looking to launch or expand mobile market operations to apply to be part of the Veggie Van study. Nine organizations were selected as partners and asked to implement the Veggie Van model at new mobile market sites and facilitate the recruitment of mobile market customers for the study and data collection activities. All study procedures were approved by the University at Buffalo Institutional Review Board and registered at www.clinicaltrials.gov (Registered on 29 January 2020) (NCT04246593).

### 2.2. Site Recruitment and Randomization

Each study partner organization was asked to partner with four community sites that serve as potential locations for the mobile market program, known as community sites. Community sites are locations where the mobile market stops and serves customers; examples include libraries, community centers, senior housing sites, etc. The community sites have not previously hosted mobile markets and were chosen based on their history of serving the study’s target population—communities experiencing lower incomes and food insecurity. Partners provided letters of support from community sites at the time of their RFP applications to demonstrate each site’s commitment. During the study’s onboarding process, partners will outline and sign a memorandum of understanding (MOU) with each community site to ensure all parties agree to the terms for operating the mobile market and engaging in research activities.

Partner organizations will choose two community sites to start working with first based on timeline and readiness. These two sites will be paired for the purposes of randomization; after MOUs are signed, one will be randomized to the mobile market site (intervention) and the other to the planning site (control). The same process will be used for the remaining pairs of sites. Partner organizations will run a mobile market following the Veggie Van model for at least 10 months at the community sites, randomized to the mobile market intervention. Planning sites will partake in a year-long food access planning process to help determine if the surrounding community will benefit from a mobile market at that location. After one year, the partner and the planning site can decide to implement a mobile market or another food access program based on community feedback gathered through the planning activities. The study team will work in tandem with the partner organizations to decide the schedule for launching the site pairs and their specific activities. Two months prior to the launch date of both the mobile market site and the planning site activities, partner organizations work jointly with community sites to engage the community to raise awareness about the mobile market and recruit potential customers to participate in the Veggie Van Study.

### 2.3. Participant Recruitment

Study participants will be recruited using a two-phase process, first directly from the partner and/or community site and then by the research team. Partner organizations and their community host sites will identify individuals who are interested in learning more about a mobile market in their community. Community engagement efforts to help with identifying community members may include community forums and listening sessions, door-to-door canvasing, informational tables at community events, and the establishment of texts, e-mails, and/or social media sites for ongoing communication and feedback around food access issues. Community sites’ programming events and social media channels will be leveraged to generate interest. To facilitate recruitment to the Veggie Van study, the partner and community site will utilize interest forms or sign-up sheets to collect community members’ contact information. These forms will also provide valuable information to help partners engage with community members on an ongoing basis and will be shared with the research team for recruitment purposes. Only if community members indicate interest in participating in the Veggie Van study will the research team then reach out to screen, provide consent, and enroll them in the study.

The goal is to recruit at least 30 participants at each of the 32 proposed mobile market sites (960 total). Eligible individuals include those who are at least 18 years old, are able to speak English and/or Spanish, are the primary grocery shoppers for their households, and live near or otherwise regularly frequent the community site. Participants will be excluded if they are planning to leave the area or stop frequenting the proposed site within the next year. To ensure that sites will be predominantly serving lower-income community members and that the study team is sampling from the appropriate target population, a goal will be set that at least 60% of eligible sign-ups are from individuals who self-report utilizing assistance programs (e.g., the Supplemental Nutrition Assistance Program (SNAP), the Special Supplemental Nutrition Program for Women, Infants, and Children (WIC), Temporary Assistance for Needy Families (TANF), Medicaid, Work First, and/or housing assistance). If the percentage is consistently below 60%, the study team and partner organization will discuss engagement strategies to better reach the intended population.

### 2.4. Intervention

The intervention is an evidence-based model intended for mobile markets, the Veggie Van (VV) model [7]. Informed by the social cognitive theory, the VV model was designed to address the multiple dimensions of access to fresh produce for lower-income and under-resourced communities: acceptability, availability, affordability, accessibility, and accommodation [9,10]. A core component of the model is offering a variety of fresh, high-quality F&V at reduced costs at convenient locations selected based on strong partnerships with organizations (community sites) that are already serving lower-income communities through complementary services (i.e., health clinics, community centers, etc.). While we do not definitely know the minimum amount of exposure to the intervention required to observe an impact, the prior Veggie Van efficacy study operated a market following the model for 6 months [7]. To maximize availability and accessibility to fresh F&V, the model recommends operating weekly for at least 10 months out of the year, but ideally year-round. A unique component of the Veggie Van model is to encourage and incentivize customers to purchase a bundle of produce (multiple items for a set price) rather than just one or two items separately. Studies investigating community-supported agriculture (CSA), which utilizes bundling, report participants eating more F&V (in number and variety) [11], increases in F&V availability, and trends of increased consumption among lower-income participants in a free CSA program [12]. The VV model also includes a cooking and nutrition education component to improve self-efficacy for finding, purchasing, and preparing F&V. Lastly, acceptance of SNAP and participation in local incentive programs further increase affordability.

### 2.5. Retention

The research team will work with each partner organization to enhance participant retention. To help maintain accurate contact information and keep participants engaged, we will collect contact information for close friends or relatives and send participants additional communication between baseline and follow-up (e.g., holiday cards and text messages). Quarterly newsletters with study updates and produce tips will be sent quarterly to all participants (intervention and control sites). As with recruitment, partner organizations and host community sites will leverage communication channels to advertise programming (either mobile markets or planning events) throughout the intervention year, as well as facilitate follow-up data collection.

### 2.6. Data Collection

**Figure 1:**
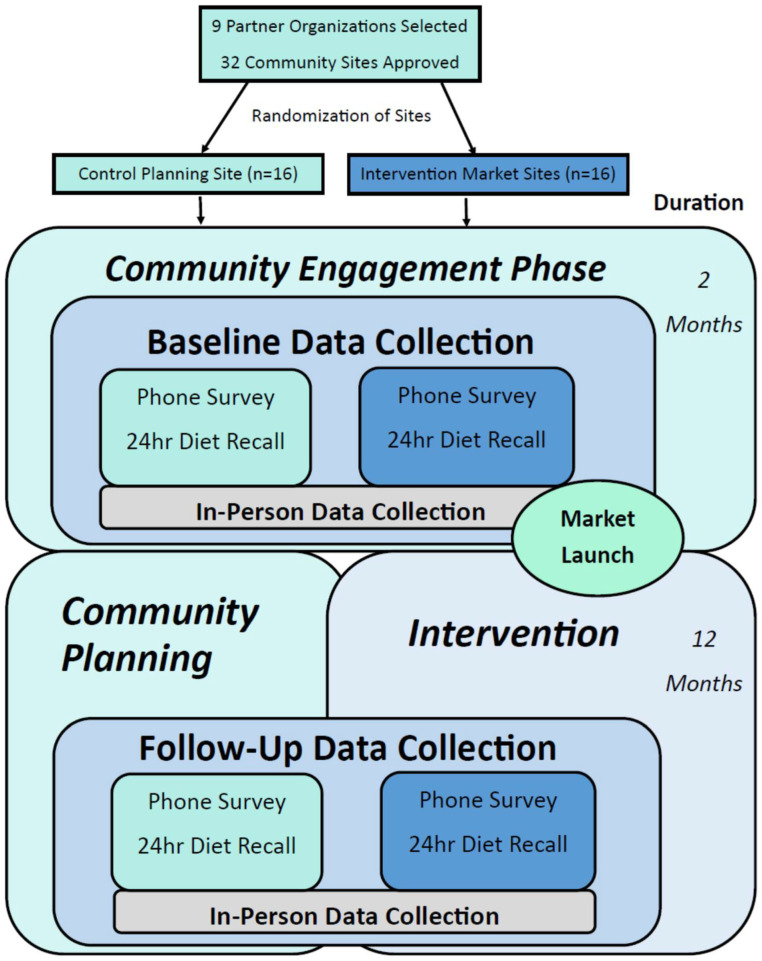
Study sequence and data collection events.

Figure 1 illustrates a flowchart of the sequence of study events, including partner selection, site randomization, and participant study activities. Data collection will take place at baseline (prior to market launch and planning activities) and at approximately 10 months later at follow-up while the market is still in operation and the planning site has not yet started a market. Data will be collected from market and planning site participants through surveys, 24-h dietary recalls, and in-person data collection events. The baseline and follow-up surveys will be administered over the phone by trained research assistants and will take approximately 30 min to complete. Survey data will be entered directly into a secure web application REDCap (Research Electronic Data Capture), supported by the University at Buffalo. Upon completion of the survey, participants will provide general availability to complete two phone-based 24-h recalls within a two-week time frame and the Food Amounts Booklet will be mailed so participants will have a visual reference during the 24-h recall.

Approximately two weeks prior to the launch of the market site (baseline) and 12 months post-market launch (follow-up), enrolled participants from both planning and market sites will be asked to attend an in-person data collection event. The study team will work jointly with the partner organization and each community site to coordinate the event. Two in-person data collection sessions will be scheduled per site to collect in-person data from all participants from that site. The event will be advertised to the community that patronizes the community site, to encourage additional enrollment and build interest in the mobile market. If participants cannot attend either session, the partner organization staff will help collect the remaining in-person data. The study team will attend and support each partner organization at the in-person data collection events, train staff on data collection procedures, and provide a detailed protocol.

Participants who complete the survey, two 24-h dietary recalls, and the in-person data collection will earn USD 45 at each time point for a total of USD 90. For community sites that do not reach 30 enrolled participants per site by the time of the market launch, recruitment will continue for up to 2 months post-market launch. However, since participants enrolled at the market site will presumably be exposed to the intervention (i.e., purchasing and eating F&V), the baseline dietary recalls will be omitted and will only complete the follow-up dietary recalls; these participants can earn up to USD 75 total (USD 30 at baseline and USD 45 at follow-up).

### 2.7. Measures

Table 1 details the participant study measures by the time point and collection method.

#### 2.7.1. Effectiveness Measures

Effectiveness measures will be collected from participants at all sites at baseline and 12 months using four methods: (1) body mass index (BMI) based on in-person height and weight measurements; (2) dermal carotenoids measured via a finger scan; (3) 24-h dietary recalls, and (4) surveys.

#### 2.7.2. BMI

BMI will be calculated at baseline and 12 months from weight measured using a SECA 876 digital scale and height measured to the nearest 1/8 inch using a SECA stadiometer. These measures will be collected during in-person events by research staff.

#### 2.7.3. Dietary Recall

Our main individual-level outcome, change in the F&V intake (cups/day) at 12 months, will be calculated from four 24-h recalls (two at baseline and two at the 12-month follow-up), which will be administered over the phone. Secondary dietary outcomes that can be calculated from these data include total calories per day, the percentage of total calories from F&V, and the percentage of total calories from added sugars. The Nutrition Data System for Research (NDSR; current version), licensed by the Nutrition Coordinating Center (NCC) at the University of Minnesota (Minneapolis, MN, USA), was used to conduct the dietary recall by phone in both English and Spanish. NDSR allows for the collection of a participant’s food intake from the previous day from midnight to midnight [13,14]. The software guides the interviewer through its completion using a dynamic user interface. Three research staff members completed a rigorous training course at the University of Minnesota on how to use the software. Ongoing monthly quality control checks of 10% of NDSRs will be scheduled to maintain the quality of food interviews and data entry. All micro- and macronutrients will be obtained, as well as data on foods and food groups. Dietary outcomes from NDSR data include total calories, fat calories, fiber, and fruit and vegetable intake [15,16]. The choice to conduct two dietary recalls was informed by past research indicating that a greater number of recalls does not increase the quality of the data; alternatively, combining this measure with an objective measure (i.e., dermal carotenoids) will reduce measurement error [17,18]. The recalls occur within a two-week time span, one recall recounts a weekday and the other a weekend. This provides a snapshot of the daily dietary intake of the participants. During the dietary recall, participants will use the Food Amounts Booklet, which will be mailed prior to recall, to help estimate how much they ate and drank. Secondary dietary outcomes, which can be calculated from these data, include total calories per day, percentage of total calories from F&V, and percentage of total calories from added sugars.

#### 2.7.4. Sales Data

Participant sales data will be collected using the Farmers Register Point-of-sale (POS) software. Farmers Register is a custom iPad-based POS system that speeds up mobile market transactions and improves tracking. Purchasing data will include the number of visits over the 12-month period as well as an itemized sales history.

#### 2.7.5. Skin Carotenoids

Skin carotenoids will be assessed using the commercially available Veggie Meter^®^ (Longevity Link, Inc., Salt Lake City, UT, USA), which relies on reflection spectroscopy to measure the level of carotenoid pigments. Emerging evidence supports the use of spectroscopy-based skin carotenoid measurements as a noninvasive objective method for determining skin carotenoid concentrations indicative of F&V intake [19,20]. We will ask participants to clean their hands with an alcohol wipe before inserting the left index finger into the meter. Each participant’s finger will be scanned 3 times and the average value will be used. A score is immediately produced via the attached computer. Scores scale linearly with tissue carotenoid levels, with higher scores indicating higher carotenoid levels. The device is calibrated before use and after one hour of continuous use or when moved.

#### 2.7.6. Phone Survey Measures

The following measures will be collected via surveys administered over the phone by trained research assistants. Demographics will be collected at baseline via the baseline survey, including age, sex, race/ethnicity, household income, marital status, education, and receipt of government assistance within the past 12 months (e.g., SNAP). Baseline and follow-up surveys will assess fruit and vegetable consumption using the 2013 Behavioral Risk Factor Surveillance System (BRFSS) questionnaire, which captures the frequency of fruit and vegetable intake over a period of 30 days. This scale reviews several food type categories, including fruit juice, fruit, beans, dark green- and orange-colored vegetables, and all other types of vegetables. Baseline and follow-up surveys will also assess shopping behaviors and attitudes, such as shopping frequency, perceptions of F&V availability, quality, and the prices at the stores that the participants usually shop at; follow-up surveys will include specific questions about the mobile market (MM perceptions). Baseline and follow-up surveys will also measure psychosocial constructs including self-efficacy, behavioral capability, outcome expectations, and observational learning. Self-efficacy to purchase, prepare, and eat fresh F&V will be measured with a 10-point Likert scale and will serve as a comprehensive assessment of the effect of the educational intervention. Benefits (expectations) and barriers to eating F&V will be measured using a 4-point Likert scale and perceived access to F&V will be measured by using three 3-item scales. This scale measures the food environment near the community host site in general and will allow us to assess the effect of the intervention on everyone (not just those who choose to opt-in by becoming customers). Data collected at 12 months will also include open-ended qualitative questions related to program satisfaction, as well as barriers and facilitators to customers using the mobile market at intervention sites.

#### 2.7.7. Implementation Measures

Implementation of the VV model will be assessed on an ongoing basis through data collection with participants (through sales data and phone measures) as well as partner organizations (through monthly process measure surveys and implementation interviews). Additional details on these measures and the analyses will be reported elsewhere in a manuscript focusing on the implementation of the VV model.

### 2.8. Sample Size

Our primary outcome will be a change in F&V intake (cups/day) at 12 months using the 24-h dietary recall; thus, we will compare the difference in mean changes in F&V between the intervention and comparison group participants. Based on our initial efficacy study [21], we estimate the intra-class correlation (ICC) for change in F&V intake to be 0.08 (SD 2.7). We expect the mobile market program to increase the F&V intake by at least 1 cup/day (effect size of approximately 0.4). Using one-sided tests of significance at *p* = 0.05, an ICC = 0.08, and cluster size = 21 participants per site, 12 sites per group will provide at least 80% power to detect the anticipated change in F&V consumption. To account for possible attrition or extended delays on the part of the organization, we will over-recruit. While 6 partner organizations (24 mobile market intervention sites) are needed to achieve the desired power, 9 organizations (32 sites) were recruited. We expect participant attrition to be no more than 30% based on previous work, so we aim to recruit at least 30 participants per site.

### 2.9. Data Analyses

Our primary analysis will test the hypothesis under the intent-to-treat principle using a generalized linear mixed model (GLMM) that will account for the correlation induced by the clustering of participants within sites. To further explore the intervention effect, we will fit GLMMs that (1) adjust for baseline covariates of interest; (2) adjust for baseline variables distributed differently between intervention groups; (3) test interaction terms between treatment group and other covariates; and (4) complete sub-group analyses. Specifically, we will look at differences in changes in F&V consumption, perceived access, and self-efficacy over time and between groups (intervention vs. control). Additionally, if we see correlations between changes in F&V consumption, self-efficacy, intervention usage, and/or perceived access at 12 months, we will conduct analyses to determine if these changes mediate the intervention effect. In addition, we will expand the regression models by including interaction terms to examine the heterogeneity of intervention and the role of moderators, such as organizations or community types. Purchasing data will be tied to participants’ survey/dietary intake data to test for correlations between dietary and sales data.

Lastly, qualitative customer feedback on the VV model will inform on improvements in community outreach and customer relations.

## 3. Discussion

This research is novel because it is the first multi-site effectiveness RCT of mobile markets in which several partner organizations across multiple states are implemented. Previous research studies on the Veggie Van model and similar mobile market programs [22,23,24] were limited by several factors. First, some were smaller, uncontrolled studies [4,21] that only looked at pre-post data within a small number of locations. Two were larger RCTs, but the intervention was run with close oversight from researchers, and the markets were only run by one community partner [5,7]. The current study also improves upon the data collection methods of the previous research. Earlier studies were limited to food frequency questionnaires and self-reported purchasing; the present study includes two 24-h dietary recalls to measure F&V intake, an imperfect gold standard for dietary data collection [25], and uses the Veggie Meter^®^ to assess dermal carotenoids, an objective measure that has been correlated with F&V intake [19,20]. We will also use a standardized point of sale (POS) software to capture purchasing across communities and mobile market programs. This software holds great potential for understanding purchasing and evaluating interventions with small markets beyond the participating mobile market organizations in this study.

The methods for this study contribute to the scant literature on how to engage in community-partnered participant recruitment. The interest form process was initially adopted as it was the preferred method used by our community partner during the first Veggie Van efficacy study [26] to ensure that there was enough interest in each community prior to starting a mobile market there. It also allowed the partner and the researcher to ensure that the demographics of the proposed community were in line with the target population (experiencing lower-income/low food access) prior to recruitment. In the previous research, we waited until enough interest forms were collected from a given community (e.g., potential mobile market community site) before they were enrolled in the study and randomized to intervention and control. Our previous research found that a common reason for the host-site dropout was that the timeline no longer worked for them as too much time was spent on interest from collection and participation recruitment that needed to occur *before* the market launch could be scheduled [26].

For the current study, we updated the process to allow community sites to join the study and be randomized prior to the collection of interest forms and/or recruitment of study participants. The proposed benefit of this approach was to limit site drop-out, which occurred due to an extended recruitment process. In the current study, the market is scheduled to open on the planned date, regardless of how many people have been recruited and we will build in some post-market launch recruitment to account for sites that are not able to reach study numbers in advance. This will allow the mobile market operator (partner) and the community (community site) to coordinate their timelines for the mobile market and start engaging in planning prior to the start of participant recruitment.

Our approach to recruitment is not without limitations. Ideally, community sites will be randomized before participants are recruited to limit selection bias. However, despite the sites knowing if they are the intervention or control at the time of participant recruitment, potential participants will still be recruited using the same language. Another limitation to the study is that not all participants will be able to complete the main outcome measure (24-h dietary recalls) at baseline since we will allow continued recruitment after the market opened. This decision was made to reduce recruitment timelines and retain partners (as described above) but may limit our ability to collect complete data; thus, it is important to include dietary measures that look at both short-term consumption (24-h recalls) and longer-term F&V consumption (food frequency questionnaires and skin carotenoids). Nonetheless, we will still be able to evaluate 12-month F&V intake (measured by the 24-h recall) for intervention communities compared to the control.

While there are both pros and cons to using a partnered approach to date collection, previous research has shown benefits, particularly for under-resourced populations. Horowitz et al. compared different recruitment strategies for a diabetes prevention intervention and the partner-led approach, in which community partners who developed and managed the recruitment efforts at their sites were the most successful and efficient at recruiting minority and lower-income individuals [27]. Reviews on recruitment and retention strategies indicate the importance of collaborating with key community leaders [28] and using community-based organizations as intermediaries [29] to effectively reach and recruit participants from diverse ethnic and cultural backgrounds.

## 4. Conclusions

The current study serves as the next step in testing the effectiveness of the VV model on a broader scale, implemented by community practitioners, and preparing to take this evidence-based model to scale [30,31,32]. If the VV model continues to demonstrate a positive impact on individual level outcomes, particularly F&V consumption, existing and future mobile markets that choose to adopt the VV model can enhance their public health impact. We are also poised to gain valuable insight into the feasibility and utility of collecting objective dietary data (i.e., Veggie Meter^®^) as well as purchasing data.

## Figures and Tables

**Table 1:** Participant study measures by the time point and collection method.

Measures	Method	Baseline (Pre-Mobile Market)	12-Month Follow-up
Height and Weight	In-Person	X	X
Dermal Carotenoids	In-Person	X	X
24-h Dietary Recall	Phone Survey	X	X
Perceived Access to F&V	Phone Survey	X	X
Shopping behavior	Phone Survey	X	X
Perceptions of grocery stores	Phone Survey	X	
Self-efficacy	Phone Survey	X	X
Demographics	Phone Survey	X	
Mobile Market Perceptions	Phone Survey		X
F&V Sales Data	POS Software	Ongoing over 12 months	

## Data Availability

The data generated during the current study will be available from the corresponding author upon reasonable request.
